# Detection of calcified carotid atheroma on panoramic dental radiography and its confirmation by Doppler ultrasound

**DOI:** 10.31744/einstein_journal/2021AI5707

**Published:** 2021-04-01

**Authors:** Breno Amaral Rocha, Leonardo de Oliveira Buzatti Carneiro, Amaro Vespasiano, Martinho Campolina Rebello Horta

**Affiliations:** 1 Pontifícia Universidade Católica de Minas Gerais Belo HorizonteMG Brazil Pontifícia Universidade Católica de Minas Gerais, Belo Horizonte, MG, Brazil.

Carotid artery atherosclerosis is one of the main causes of stroke.^(1)^ In dental panoramic radiography (DPR), radiopaque images adjacent to cervical spine can be an indicative of calcification in bifurcation of carotid artery. Such calcifications can represent calcified atheroma plaques,^(2)^ which diagnosis is confirmed by the Doppler ultrasonography.^(3,4)^

Dentists can identify these areas through DPR and differentiate them from radiopacities that can be observed in the carotid region. The identification of these area and referral of these patients to specialized medical evaluation may contribute to stroke prevention.^(1,3,5)^

**Figure 1:**
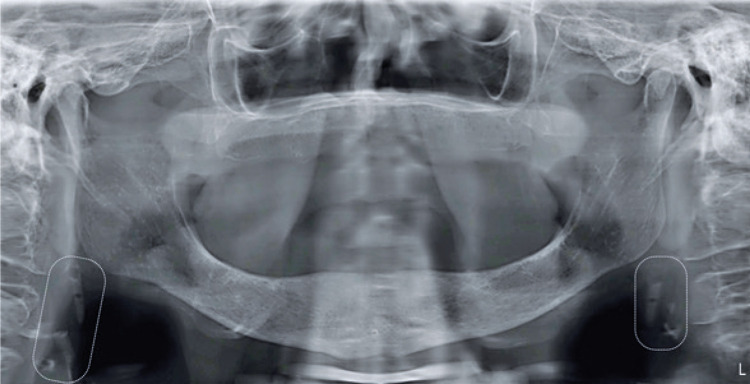
Panoramic radiography presenting areas of calcifications in soft tissue at level of vertebrae C3 and C4, bilaterally

**Figure 2:**
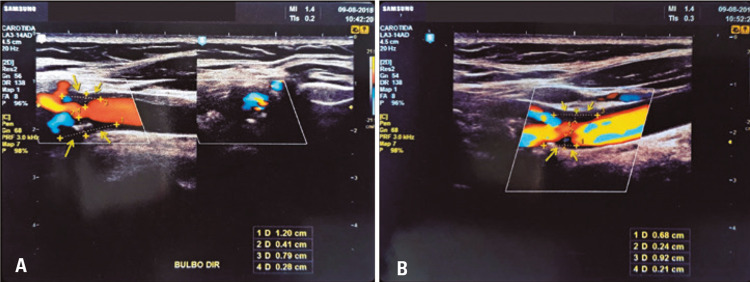
Doppler ultrasonography images (A and B) presenting atheromatous plaques (indicated by the arrows) in topography of carotid bifurcation on the right and left side, respectively

We report a case of 86-year woman admitted to our diagnostic oral service complaining of oral pain. At oroscopy, we did not observe lesions. Therefore, we requested a RPO which did not reveal changes associated with the patient’s main complaint. However, we observed diffuse calcifications in proximity to vertebrae C3 and C4, bilaterally (Figure 1). Based on these findings, the hypotheses were calcified carotid atheroma, triticeous cartilage, and lymph node calcifications. Due to the hypothesis of calcified carotid atheromas, we requested a color pulsed Doppler ultrasonography. Results showed common carotid with increased intima-media thickness, carotid bulb with atheromatous plaque with stenosis lower than 50% (Figures 2A and 2B), and internal carotid with atheromatous plaque with stenosis signs between 50% and 69% on both sides. The patient was referred to cardiological evaluation, and currently she is under medical supervision. We highlight the importance of observe radiopacities on carotid artery topography through DPR and the posterior diagnostic definition for stroke prevention.
