# Supplementary material for: Detection of calcified carotid atheroma on panoramic dental radiography and its confirmation by Doppler ultrasound
Source: Einstein (Sao Paulo). 2021 Apr 1;19:eAI5707. doi: 10.31744/einstein_journal/2021AI5707 (PMC8020837; doi:10.31744/einstein_journal/2021AI5707)
Supplement: Translation

# Translation: Detecção de ateroma calcificado de carótida em radiografia panorâmica odontológica e sua confirmação por meio de ultrassonografia com Doppler

**Authors:** Breno Amaral Rocha, Leonardo de Oliveira Buzatti Carneiro, Amaro Vespasiano, Martinho Campolina Rebello Horta

**Affiliations:** 1 Brasil Pontifícia Universidade Católica de Minas Gerais, Belo Horizonte, MG, Brasil.

A aterosclerose da artéria carótida é uma das principais causas do acidente vascular cerebral (AVC).^(1)^ Em radiografia panorâmica odontológica (RPO), imagens radiopacas adjacentes à coluna cervical podem ser indicativas de calcificações na bifurcação da artéria carótida. Elas podem representar ateromas calcificados,^(2)^ cuja confirmação diagnóstica é feita por meio da ultrassonografia com Doppler.^(3,4)^ Cirurgiões-dentistas podem identificar essas áreas em RPO e diferenciá-las de radiopacidades que podem ser observadas na região carotídea. A identificação dessas áreas e o encaminhamento desses indivíduos a uma avaliação médica especializada pode contribuir para a prevenção do AVC.^(1,3,5)^

Relata-se o caso de uma paciente, do sexo feminino, 86 anos, que compareceu ao serviço de diagnóstico oral para avaliação de dor bucal. À oroscopia, não foram observadas lesões. Solicitou-se, então, uma RPO, na qual não foram observadas alterações associadas à queixa principal. Entretanto, observaram-se calcificações difusas na proximidade das vértebras C3 e C4, bilateralmente (Figura 1). Diante desses achados, as hipóteses foram ateroma calcificado em carótida, cartilagem tritícea e linfonodos calcificados. Em virtude da hipótese de ateroma calcificado nas carótidas, solicitou-se ultrassonografia com Doppler colorido e pulsado que demonstrou carótidas comuns com espessura mediointimal aumentada, bulbos carotídeos com placas ateromatosas com estenose menor que 50% (Figuras 2A e 2B) e carótidas internas com placas ateromatosas com sinais de estenose entre 50% a 69%, bilateralmente. A paciente foi encaminhada para avaliação cardiológica e encontra-se em acompanhamento médico. Portanto, destaca-se a importância da observação de radiopacidades na topografia da artéria carótida em RPO e sua posterior definição diagnóstica, visando à prevenção do AVC.

**Figura 1:** Radiografia panorâmica apresentando áreas de calcificações em regiões de tecido mole, na altura das vértebras C3 e C4, bilateralmente

**Figura 2:** Imagens (A e B) ultrassonográficas com Doppler apresentando placas ateromatosas (indicadas pelas setas) na topografia da bifurcação carotídea à direita e à esquerda, respectivamente
